# Unraveling the *Sclerotinia* Basal Stalk Rot Resistance Derived From Wild *Helianthus argophyllus* Using a High-Density Single Nucleotide Polymorphism Linkage Map

**DOI:** 10.3389/fpls.2020.617920

**Published:** 2021-02-03

**Authors:** Zahirul I. Talukder, William Underwood, Christopher G. Misar, Gerald J. Seiler, Yuan Liu, Xuehui Li, Xiwen Cai, Lili Qi

**Affiliations:** ^1^Department of Plant Sciences, North Dakota State University, Fargo, ND, United States; ^2^United States Department of Agriculture – Agricultural Research Service, Edward T. Schafer Agricultural Research Center, Fargo, ND, United States

**Keywords:** sunflower, introgression, *Helianthus argophyllus*, *Sclerotinia sclerotiorum*, basal stalk rot resistance, quantitative trait loci

## Abstract

Basal stalk rot (BSR), caused by the fungus *Sclerotinia sclerotiorum*, is a serious disease of sunflower (*Helianthus annuus* L.) in the humid temperate growing areas of the world. BSR resistance is quantitative and conditioned by multiple genes. Our objective was to dissect the BSR resistance introduced from the wild annual species *Helianthus argophyllus* using a quantitative trait loci (QTL) mapping approach. An advanced backcross population (AB-QTL) with 134 lines derived from the cross of HA 89 with a *H. argophyllus* Torr. and Gray accession, PI 494573, was evaluated for BSR resistance in three field and one greenhouse growing seasons of 2017–2019. Highly significant genetic variations (*p* < 0.001) were observed for BSR disease incidence (DI) in all field screening tests and disease rating and area under the disease progress curve in the greenhouse. The AB-QTL population and its parental lines were genotyped using the genotyping-by-sequencing method. A genetic linkage map spanning 2,045.14 cM was constructed using 3,110 SNP markers mapped on 17 sunflower chromosomes. A total of 21 QTL associated with BSR resistance were detected on 11 chromosomes, each explaining a phenotypic variation ranging from 4.5 to 22.6%. Of the 21 QTL, eight were detected for BSR DI measured in the field, seven were detected for traits measured in the greenhouse, and six were detected from both field and greenhouse tests. Thirteen of the 21 QTL had favorable alleles from the *H. argophyllus* parent conferring increased BSR resistance.

## Introduction

Sunflower (*Helianthus annuus* L.) ranks third in worldwide production among the important vegetable oil-producing crops after soybean and rapeseed (USDA, 2020). Sunflower was grown on 26.7 million ha worldwide in 2018 producing about 52 million metric tons of sunflower seeds (FAOSTAT, 2020). The sunflower industry has a significant effect on the agricultural economy of the northern and central Great Plains of the United States, with an annual acreage of approximately 0.8 M ha (Gulya et al., 2019). One of the most important constraints to sunflower production is the disease caused by the necrotrophic fungal pathogen, *Sclerotinia sclerotiorum* (Lib) de Bary. The fungus has a very broad host range with over 400 dicotyledonous plant species, including some important food crops like soybean, canola, sunflower, dry beans, peas, and other pulse crops (Boland and Hall, 1994). *S. sclerotiorum* is a devastating pathogen of sunflower that can cause infection at any growth stage (Gulya et al., 1997). A root infection initiated by myceliogenic germination of sclerotia (unique to sunflower) causes basal stalk rot (BSR) and wilt, while carpogenic germination of sclerotia releases airborne ascospores and causes midstalk rot (MSR) by infecting sunflower leaves or head rot (HR) by infecting capitula (Gulya et al., 1997). All these diseases can cause serious economic damage to sunflower by both yield loss and reduced seed quality.

*Sclerotinia* BSR disease is the most damaging in the humid temperate Northern Great Plains (North Dakota, South Dakota, and Minnesota) where most of the United States sunflower is grown. Fungicidal control of BSR disease is not feasible because the infection begins belowground (Talukder et al., 2019a). The use of host resistance is the most economic and environmentally friendly option for BSR management in sunflower. The genetics of BSR resistance is quantitative in nature and governed by multiple small effect genes (Talukder et al., 2014c). Breeding for BSR resistance traditionally followed the multiparent crosses of partially tolerant lines available in the primary gene pool followed by recurrent selection to achieve increased *Sclerotinia* resistance. Several sunflower germplasms and inbred lines with improved *Sclerotinia* resistance have been selected and released by the USDA-ARS for use in sunflower hybrid development (Miller and Gulya, 1999; Miller et al., 2006; Talukder et al., 2017; Hulke et al., 2018; Money et al., 2019; Smart et al., 2019).

The estimation of the effects and positions of quantitative trait loci (QTL) is of central importance in dissecting and efficiently utilizing quantitative trait variation in a breeding program. Few QTL mapping studies of BSR resistance have been reported in sunflower. A recombinant inbred line (RIL) population derived from the cross of PAC2/RHA266 was assessed for *Sclerotinia* BSR resistance under a controlled environment in growth chambers (Davar et al., 2010; Amouzadeh et al., 2013). Davar et al. (2010) screened 116 RILs with *S. sclerotiorum* isolate SSU107 and identified seven QTL for percentage necrotic area associated with BSR resistance localized on linkage groups (LGs) 1, 2, 4, 6, 8, 14, and 17, while Amouzadeh et al. (2013) assessed 99 RILs of the same population with the fungal isolate SSKH41 and identified five QTL on LGs 1, 3, 8, 10, and 17. The estimated effects of QTL in both studies were small (explaining 0.5–8% of total variation each), confirming polygenic control of the trait. These mapping studies were conducted by inoculating plants with *S. sclerotiorum* mycelium at the base of the stem. The lack of correlation between *Sclerotinia* head rot and BSR resistance suggests that sunflower resistance to *S. sclerotiorum* is likely to be tissue specific, and consequently, it is unclear if basal stem inoculation accurately predicts root resistance to BSR (Masirevic and Gulya, 1992; Talukder et al., 2014b). A RIL population derived from the cross of inbred lines HA 441/RHA 439 was evaluated for *Sclerotinia* BSR resistance in North Dakota and Minnesota over multiple years (Talukder et al., 2016). A total of six BSR resistance QTL were identified, one each on LGs 4, 9, 10, 11, 16, and 17. The most significant QTL on LGs 10 and 17 were identified from multiple environments that explained about 31.6 and 20.2% of the observed phenotypic variance, respectively. A candidate gene association mapping study using 260 sunflower lines revealed that the candidate genes *HaCOI1*-*1* and *HaCOI1*-*2* on sunflower LG14, orthologous and paralogous to the *Arabidopsis thaliana COI1* gene, respectively, were strongly associated with *Sclerotinia* BSR resistance and explained 7.4% of phenotypic variation in this population (Talukder et al., 2014c). The *HaCOI1*-*1* gene was also reported to be associated with *Sclerotinia* HR resistance (Filippi et al., 2020).

Crop wild relatives (CWR) are a valuable source of novel genes lost during the domestication process. The genus *Helianthus* encompasses 53 species, including 14 annual and 39 perennial (Seiler et al., 2017). Annual *Helianthus* species are all diploid (2*n* = 2*x* = 34). Wild annual *Helianthus argophyllus*, commonly known as “silverleaf sunflower” is native to the coastal regions of Texas and some neighboring states of the United States. Genes for several economically important traits have been reported in *H. argophyllus* including resistance to downy mildew, *Sclerotinia*, and *Phomopsis*, as well as genes controlling cytoplasmic male sterility and fertility restoration (for review, see Qi et al., 2016a; Ma et al., 2017; Seiler et al., 2017). A recent pan-genomic analysis revealed that 4.7% of the gene content of the cultivated sunflower primary gene pool is derived from wild *H. argophyllus* (Hubner et al., 2019).

Block et al.(2008, 2009, 2010) identified *H. argophyllus* accessions highly resistant to *Sclerotinia* BSR in the USDA gene bank collection using an innovative greenhouse screening technique. Later, Qi et al. (2016b) transferred BSR resistance from a selected *H. argophyllus* accession into a cultivated sunflower background, and germplasm lines possessing elevated levels of BSR resistance have been released for use in sunflower BSR resistance breeding (Qi et al., 2018).

Despite reports of high levels of BSR resistance in *H. argophyllus*, CWR are also known to carry undesirable traits often linked with the gene of interest, thereby making its use extremely difficult. To overcome such problems in interspecific gene transfer, Tanksley and Nelson (1996) proposed an “advanced backcross QTL (AB-QTL)” method to identify and transfer the desired alleles from a wild donor into the cultivated background. In this method, QTL analysis is conducted in a backcross population and the frequency of deleterious donor alleles is reduced by negative selection during population development (Tanksley and Nelson, 1996).

Phenotypic backcross selection for introgression of desirable genes from wild relatives like *H. argophyllus* is labor- and cost-intensive and may also encounter linkage drag for other traits. This research aims to map QTL associated with *Sclerotinia* BSR resistance segregating in an AB-QTL mapping population developed from the cross of a highly BSR-resistant *H. argophyllus* accession with a cultivated sunflower line. This is, so far, the first report known to map BSR resistance derived from a sunflower CWR. This research will enhance our understanding of the molecular basis of *Sclerotinia* BSR resistance existing in CWR and benefit sunflower molecular breeding to improve *Sclerotinia* BSR resistance.

## Materials and Methods

### Plant Materials

An AB-QTL mapping population comprised of 134 progeny lines (BC_1_F_2_ for genotyping and BC_1_F_2_-derived BC_1_F_4_ for phenotyping) was developed by crossing the cultivated sunflower line HA 89 with a wild annual *H. argophyllus* accession (PI 494573). The initial cross was made with the nuclear male sterile (NMS) HA 89 (PI 559477), while backcrossing was conducted with the normal, male-fertile HA 89 (PI 599773). HA 89 is an oilseed maintainer inbred line highly susceptible to BSR disease. NMS HA 89 was developed in 1990 by inducing male sterility in HA 89 using streptomycin treatment and possesses a recessive nuclear male sterility gene, *ms9* (Jan and Rutger, 1988; Chen et al., 2006). The wild *H. argophyllus* accession (PI 494573) is a diploid (2*x* = 2*n* = 34) collected from Texas and exhibits high levels of BSR resistance (Block et al., 2008, 2009, 2010).

### Experimental Design and Phenotypic Evaluation

#### Field Evaluation

The AB-QTL population along with the recurrent parent HA 89 was screened for BSR resistance in a field nursery in Carrington, ND, United States, during three growing seasons from 2017 to 2019. The design of the field trials was a randomized complete block with three replications. Thirty seeds of each line were planted in a 6-m-long single-row plot with 75-cm spacing between rows. The commercial sunflower hybrids ‘Cargill 272’ and ‘Croplan 305’ were used as susceptible and resistant check, respectively. Five to six weeks after planting, at the V-6 growth stage (Schneiter and Miller, 1981) when plants are <30 cm tall, approximately 90 g of proso millet (*Panicum miliaceum* L.) infested with *S. sclerotiorum* mycelium (isolate, NEB-274) were placed in furrows 15–20 cm from the rows at a depth of 5 cm using a tractor-drawn granular chemical applicator (Gulya et al., 2008).

#### Greenhouse Evaluation

The AB-QTL population was evaluated in the greenhouse in three replications in the winter of 2019 as described by Underwood et al. (2020). Initially, 20 seeds of each of the progeny lines along with the seeds of the recurrent parent, HA 89, and a resistant check, RHA 801, were grown in 3-inch deep sheet pots (T.O. plastics, Clearwater, MN, United States) of 21.06-inch × 10.56-inch dimensions each containing eight rows of four wells. Sheet pots were filled with potting mix (Metro Mix 902, Sun Gro Horticulture, Agawam, MA, United States) with a single sunflower seed in each well and allowed to grow in the greenhouse at ∼22°C temperature with 16 h photoperiod provided by supplemental lighting. After 5 weeks, 12 seedlings of each progeny line, parent, and check were inoculated with *S. sclerotiorum* (isolate NEB-274)-infested millet. Inoculations were performed by carefully removing the plants from the sheet pot wells without disturbing the root mass and placing a one-half teaspoon scoop (∼0.76 g) of inoculum at the bottom of the well and returning the plant back into the well to sit on top of the inoculum. The inoculated plants were then randomly arranged in the greenhouse in 12 blocks and grown for another 4 weeks for data collection.

### Data Collection and Statistical Analysis

In the field screening trials, disease incidence (DI) was determined at maturity in each row as the percentage of plants showing BSR lesions at the soil line. In the greenhouse screening trials, plant death due to BSR was monitored and recorded daily for 28 days post-inoculation (dpi), and days-to-death data were used to determine area under the disease progress curve (AUDPC) and BSR disease rating (DR). Disease rating is measured as the average number of days required to show terminal wilting or whole-plant desiccation that ultimately results in the death of the inoculated plant. Plants that remained alive and showed normal growth at 28 dpi were assigned a maximum value of 29. AUDPC was calculated as:

$$\sum_{i=1}^{n-1}\frac{\left(y_{i}+y_{i+1}\right)}{2}\,\left(t_{i+1}-t_{i}\right)$$

where *y*_*i*_ is the proportion of inoculated plants dead due to BSR at the *i*th observation, *t* is the time (dpi) at the *i*th observation, and *n* is the total number of observations *(Shaner and Finney, 1977)*.

The distribution of both field and greenhouse BSR data was first checked for normality using the Shapiro–Wilk normality test. Prior to performing the combined analysis for multiple years field data, homogeneity of variance was confirmed using Levene’s test. The data were then analyzed in SAS v9.4 *(SAS Institute, 2016)* using a generalized linear mixed model (Proc GLIMMIX) to determine significant differences for DI among progeny lines. Genotype was considered as fixed effect, while year and replication were considered as random effects in the model. Broad-sense heritability (*H*^2^) was estimated on an entry mean basis for BSR data measured in both field and greenhouse following Nyquist (1991):

(i) $$H^{2}=\frac{\sigma_{g}^{2}}{\left(\sigma_{g}^{2}+\sigma_{g\,e}^{2}/l+\sigma_{e}^{2}/l\,r\right)}$$

(ii) $$H^{2}=\frac{\sigma_{g}^{2}}{\left(\sigma_{g}^{2}+\sigma_{e}^{2}/r\right)}$$

The equation (i) is for BSR DI measured in the field, and equation (ii) is for AUDPC and DR measured in the greenhouse, where $\sigma_{g}^{2}$ is the genotypic variance, $\sigma_{g\,e}^{2}$ is the genotype × environment variance, $\sigma_{e}^{2}$ is the error variance, *r* is the number of replications, and *l* is the number of environments. Spearman’s rank correlation among field and greenhouse BSR data was carried out using statistical package R v3.4.3 (R Core Team, 2017).

### DNA Extraction and Single Nucleotide Polymorphism Genotyping

The AB-QTL population along with the parents was gentoyped using the genotyping-by-sequencing (GBS) method. The seedlings were grown in the greenhouse in 36-well plastic sheet pots placed on trays filled with ProMix BX potting media (Premier Horticulture Inc., Quakertown, PA, United States). After 3–4 weeks of planting, leaf tissues were collected from young seedlings of each BC_1_F_2_ individual and freeze-dried. A Qiagen DNeasy 96 plant kit (Qiagen, Valencia, CA, United States) was used to extract genomic DNA from ∼50 mg of freeze-dried leaf tissue per line with a minor modification of the manufacturer’s protocol following *Horne et al. (2004)*. The isolated DNA was quantified with a Quant-iT PicoGreen dsDNA assay kit (Thermo Fisher Scientific, Waltham, MA, United States). GBS libraries were constructed based on the protocol of *Elshire et al. (2011)* with minor modifications. Briefly, 100 ng of each DNA was digested with *ECOT22I* and then ligated to a barcoded adapter unique to each sample and a common adapter. Equal volumes of the ligated products were pooled and purified with the QIAquick PCR purification kit (Qiagen, Valencia, CA, United States) for PCR amplification. For the PCR amplification, 50 ng of template DNA was mixed with NEB 2X Taq Master Mix and two primers (5 nmol each) in 200 μl of total volume and amplified on a thermocycler for 18 cycles with 10 s of denaturation at 98°C, followed by 30 s of annealing at 65°C, and finally 30 s extension at 72°C. The PCR product was then cleaned using a QIAquick PCR purification kit. The library was sequenced on an Illumina HiSeq 2500 (Illumina, San Diego, CA, United States) to generate single-end, 100-bp reads at the Genomic Sequencing and Analysis Facility at the University of Texas Southwestern Medical Center at Dallas, Texas. Single nucleotide polymorphism (SNP) discovery and genotype calling were performed using TASSEL-GBS v2 Pipeline *(Glaubitz et al., 2014)*. Sequences were condensed into unique tags and mapped to the reference genome HanXRQr1.0 *(Badouin et al., 2017)* using Bowtie2 *(Langmead and Salzberg, 2012)*. A total of 63,058 SNP markers were obtained.

### Linkage Mapping

The SNP markers were further filtered from the array of 63,058 SNP markers following the criteria: (i) all monomorphic SNP markers were removed, (ii) SNP markers having missing genotype for the recurrent parent were removed, (iii) SNP markers with more than 20% missing genotype were removed, and iv) highly distorted SNP markers from the expected ratio of 1:2:1 (*p* > 0.05) were removed. A total of 3,500 filtered SNP markers were used to construct the linkage map of the AB-QTL population using JoinMap 4.1 software (Stam, 1993; Van Ooijen, 2006). In JoinMap, the SNP markers were first analyzed to identify co-segregating markers with identical genotype using the “similarity of loci” option of the software. Prior to linkage analysis, similar loci were excluded to reduce the burden of analysis, while the remaining proxy SNP markers were assigned to linkage groups applying the independence logarithm of the odds (LOD) parameter with LOD threshold values ranging from 3 to 10. In JoinMap, the regression mapping algorithm option was used to perform linkage analysis and marker order where recombination fractions were converted to map distances in centimorgans (cM) using the Kosambi mapping function (Kosambi, 1943). Seventeen LGs corresponding to the 17 sunflower chromosomes were constructed using the SNP markers. The excluded co-segregating similar loci markers were put back into the final map in their corresponding positions.

### QTL Mapping

Initial QTL analysis was performed in WinQTL Cartographer v2.5 software by choosing the composite interval mapping (CIM) option (Zeng, 1994; Wang et al., 2012). QTL analysis of BSR field DI was analyzed using data from individual years as well as a combined analysis using genotype BLUP (best linear unbiased predictor) extracted across all 3 years. Non-normal data were transformed prior to QTL analysis using the Box–Cox transformation (Box and Cox, 1964) in R software v3.4.3 (R Core Team, 2017) of the MASS package (Venables and Ripley, 2002). In the CIM analysis, the QTL scan was performed across the sunflower genome by choosing model 6 option that accounted for both the forward and backward regression method. The scan option of the program was optimized for a window size of 10 cM with a walk speed of 1 cM and to select up to five control markers for the analysis. Significance LOD threshold values were determined independently using 1,000 times permutation tests (Churchill and Doerge, 1994). QTL analyses were also performed with other popularly used software including QGene v4.3 (Joehanes and Nelson, 2008), PLABQTL v1.2 (Utz and Melchinger, 2006), and QTL IciMapping v4.1 (Meng et al., 2015), and the results were compared with those of WinQTL Cartographer. These software packages provide similar or different algorithms and/or options for cofactor selection. Significant QTL that were repeatedly detected in the same genomic region with at least two software packages were reported in the present study. A 95% confidence interval was used to estimate the QTL flanking region using 1-LOD of the most likely QTL peak position. MapChart v2.2 (Voorrips, 2002) was used to depict the linkage map along with the detected QTL. Naming of the BSR resistance QTL followed the convention proposed by Talukder et al. (2016), with a prefix Q followed by a three-letter descriptor of the phenotype, the LG number, and a serial number.

### Sequence Retrieval of the Significant SNP Markers

A 400-bp nucleotide sequence flanking each significant BSR resistance QTL was retrieved from the XRQr1.0 sunflower reference genome assembly^1^ (Badouin et al., 2017). The detail of these significant SNP markers along with the retrieved sequences is presented in Supplementary Table 1.

## Results

### BSR Disease Screening in the Field

**FIGURE 1:**
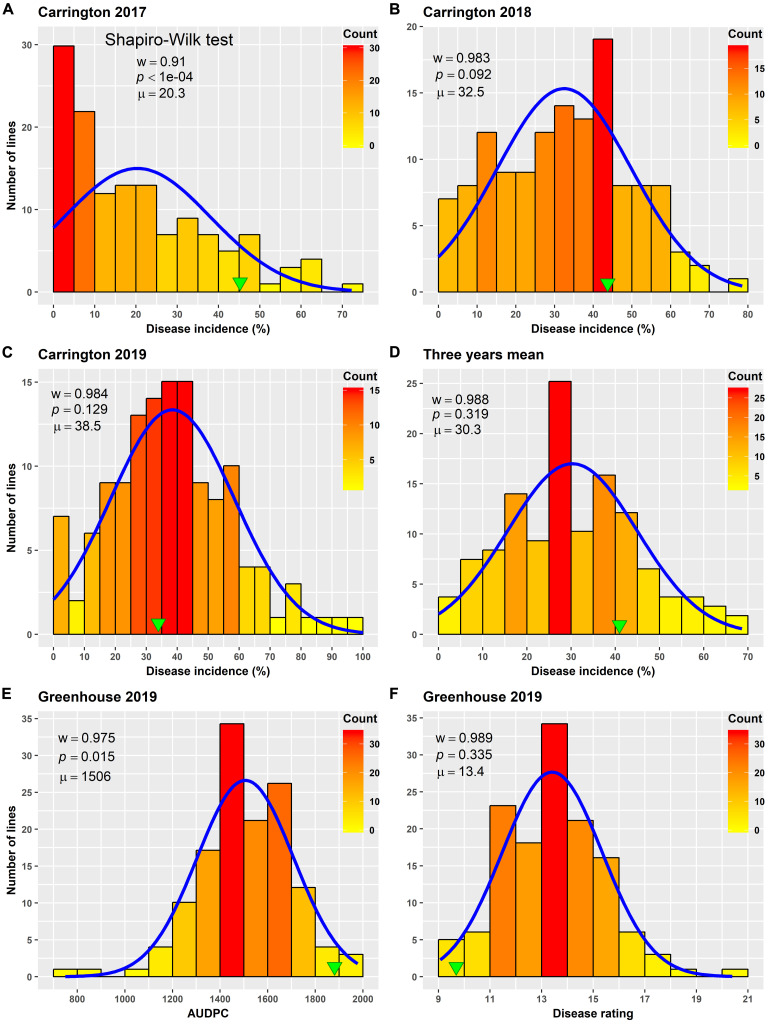
Frequency distribution of *Sclerotinia* basal stalk rot disease incidence **(A–D)**, area under disease progress curve **(E)**, and BSR disease rating **(F)** in an AB-QTL population evaluated in the field in Carrington, ND, United States during 2017 to 2019 **(A–D)** and in the greenhouse in 2019 **(E,F)**. The green arrowheads indicate the value of the recurrent parent HA 89. The Shapiro–Wilk normality test statistic (*w*), the probability value (*p*), and the mean (*μ*) of the data for each environment are shown inside the respective plots.

Varying levels of BSR DI were observed in the field screening trials of the AB-QTL population conducted in Carrington, ND, during the summer of 2017–2019 (Figure 1 and Supplementary Figure 1). The mean BSR DI for the AB-QTL population across 3 years was 30.3% and ranged from 0 to 68.7%. Overall, the distributions of the BSR DI within the population were continuous, a characteristic of the typical quantitative disease resistance (Figure 1). Although the distributions of BSR DI data were normal for 2018 and 2019 and for the 3-years’ mean DI, it was skewed toward lower DI values in the 2017 season (Figure 1). The BSR DI of the recurrent parent HA 89 for the 2017, 2018, and 2019 seasons and for the mean across the 3 years were 45.2, 43.7, 33.9, and 41.0%, respectively (Figure 1). Analysis of variance (ANOVA) showed highly significant (*p* < 0.001) genetic variations for BSR DI in all the 3 years. In the combined analysis, only the genotypes showed significant variation for the trait with no significant effect of the environment or the genotype × environment interaction (Table 1). The entry mean basis of the broad-sense heritability (*H*^2^) estimates of the BSR DI in the field across the 3 years was 0.74. Spearman’s rank correlations (*ρ*) among BSR DI data across the 3 years of field screening were highly significant (*p* < 0.001), suggesting a high level of repeatability of the BSR screening across years in the field (Table 2).

**TABLE 1:** Combined analysis of variance for *Sclerotinia* basal stalk rot disease incidence scores among lines of the HA 89/*H. argophyllus* AB-QTL population tested in Carrington, ND, during 2017 to 2019.

Component	*df*	Variance estimate	Confidence limit (0.05)		*F*/*Z* value^†^	*p* value > *F*/*Z*
			Lower	Upper		
Genotype (Gen)	133	$\sigma_{g}^{2}$ = 156.71	13.98	222.56	**3.73**	**<0.0001**
Environment (Env)	2	$\sigma_{i}^{2}$ = 71.42	16.66	10,635	0.84	0.2002
Rep (Env)	6	$\sigma_{r}^{2}$ = 36.12	13.90	211.64	1.57	0.0587
Gen × Env	264	$\sigma_{g\,i}^{2}$ = 0.81	0.38	1.81E165	0.10	0.4610
Error	779	$\sigma_{e}^{2}$ = 500.38	453.52	553.16	19.75	<0.0001

**F*, Fisher’s *F*-test statistic; *Z*, *Z*-test statistic. ^†^In the PROC MIXED model, genotypes were considered fixed and, therefore, subject to *F*-test (values in bold).*

**TABLE 2:** Spearman rank correlations (*ρ*) between *Sclerotinia* basal stalk rot disease data among lines of the HA 89/*H. argophyllus* AB-QTL population tested in the field and in the greenhouse during 2017 to 2019.

Environment		Carrington 2017	Carrington 2018	Carrington 2019	AUDPC
Field	Carrington 2017	−	−	−	−
	Carrington 2018	0.48***	−	−	−
	Carrington 2019	0.50***	0.46***	−	−
Greenhouse	AUDPC	0.11	0.09	0.18*	−
	Disease rating	0.11	0.08	0.16	0.98***

*Significant at the 0.05 probability level, ***significant at the 0.001 probability level.

### BSR Disease Screening in the Greenhouse

ANOVA analysis revealed highly significant (*p* < 0.001) genetic variation for AUDPC and DR measured in the AB-QTL population in the greenhouse trials (Table 3). The distribution of AUDPC data across three replications was continuous but somewhat skewed (*p* < 0.05) toward higher values (Figure 1). The mean AUDPC across the population was 1,506, ranging from 750 to 1,978 with the recurrent parent HA 89 mean of 1,881. The distribution of DR data was normal with a mean DR score of 13.4 for the population. The DR scores ranged from 9.1 to 20.4 with recurrent parent HA 89 score of 9.7 (Figure 1). The broad-sense heritability (*H*^2^) estimate for both AUDPC and DR was 0.73. Spearman’s rank correlations (*ρ*) between AUDPC and DR scores among the progeny lines of the AB-QTL population were highly significant (*ρ* = 0.98, *p* < 0.001) (Table 2).

**TABLE 3:** Analysis of variance for area under disease progress curve (AUDPC) and *Sclerotinia* basal stalk rot disease rating (DR) scores measured among lines of the HA 89/*H. argophyllus* AB-QTL population tested in the greenhouse.

Trait	Component	*df*	Variance estimate	Confidence limit (0.05)		*F*/*Z* value^†^	*p* value > *F*/*Z*
				Lower	Upper		
AUDPC	Genotype	133	$\sigma_{g}^{2}$ = 29,947	21,993	43,178	**3.71**	**<0.0001**
	Rep	2	$\sigma_{r}^{2}$ = 82.50	7.50	4.354E+26	0.25	0.4014
	Error	266	$\sigma_{e}^{2}$ = 33,127	28,146	39,565	11.53	<0.0001
DR	Genotype	133	$\sigma_{g}^{2}$ = 2.764	2.0272	3.9923	**3.69**	**<0.0001**
	Rep	2	$\sigma_{r}^{2}$ = 0.007	0.0006	1.932E+26	0.23	0.3994
	Error	266	$\sigma_{e}^{2}$ = 3.068	2.6029	3.6612	11.50	<0.0001

**F*, Fisher’s *F*-test statistic; *Z*, *Z*-test statistic. ^†^ In the PROC MIXED model, genotypes were considered fixed and, therefore, subject to *F*-test (values in bold).*

### Linkage Map Construction

Linkage analysis mapped a total of 3,110 SNP markers to 1,774 unique loci on 17 LGs corresponding to 17 sunflower chromosomes (Table 4). A detailed description of the HA 89/*H. argophyllus* AB-QTL population linkage map is shown in Supplementary Table 2. Approximately, 43% (1,336 SNPs) of the total mapped SNP markers co-segregated with other SNPs in the linkage groups. The largest number of mapped loci was on LG5 (256), followed by LG2 (197), LG7 (192), LG1 (173), and LG8 (162). All these LGs had over 100 loci mapped in the linkage analysis. The lowest number of loci was mapped in LG6 (35), which also had the most sparsely distributed loci (3.64 cM/locus) and markers (1.82 cM/marker). Among the mapped SNPs, a total of 524 (∼17%) markers had a distorted segregation ratio. The highest number of distorted markers mapped to LG16 (49%), followed by LG3 (43%) and LG4 (42%). None of the markers mapped in LG5 were distorted from the expected segregation ratio. The total length of the linkage map covered 2,045.14 cM with an average of 1.15 cM^–1^ locus and 0.66 cM^–1^ marker across the sunflower genome (Table 4). The length of individual LGs ranged from 97.69 cM for LG8 to 168.23 cM for LG9. In the linkage map, most of the gaps between two adjacent loci were smaller, with 95.8% (1,683 of 1,757) of the gaps being less than 5 cM (Table 4 and Supplementary Table 2). There were 13 gaps > 10 cM in the linkage map with the largest being 19.85 cM on LG6.

**TABLE 4:** Summary of sunflower linkage map developed using SNP markers in an advanced backcross population derived from the cross of HA 89 and wild annual sunflower species, *H. argophyllus.*

Linkage group	Map length (cM)	No. of loci	No. of markers	No. of distorted markers	cM/locus	cM/marker	No. of large gaps	
							5–10 cM	>10 cM
LG1	129.070	173	313	64	0.75	0.41	4	1
LG2	121.964	197	342	59	0.62	0.36	4	0
LG3	149.527	74	136	58	2.02	1.10	5	2
LG4	117.342	79	143	60	1.49	0.82	3	1
LG5	111.069	256	463	0	0.43	0.24	2	0
LG6	127.512	35	69	8	3.64	1.82	5	2
LG7	114.887	192	334	75	0.60	0.34	4	1
LG8	97.693	162	290	1	0.60	0.34	3	1
LG9	168.226	88	165	36	1.91	1.02	5	0
LG10	104.968	97	180	21	1.08	0.58	2	0
LG11	99.588	64	110	17	1.56	0.91	1	1
LG12	105.481	61	100	9	1.73	1.05	2	1
LG13	102.488	56	80	11	1.83	1.28	3	0
LG14	115.521	60	118	15	1.93	0.98	6	0
LG15	130.686	72	100	27	1.82	1.31	3	1
LG16	147.530	58	101	49	2.54	1.46	6	1
LG17	101.589	50	66	14	2.03	1.54	3	1
Total	2,045.141	1,774	3,110	524	1.15	0.66	61	13

### Quantitative Trait Loci Analysis

**FIGURE 2:**
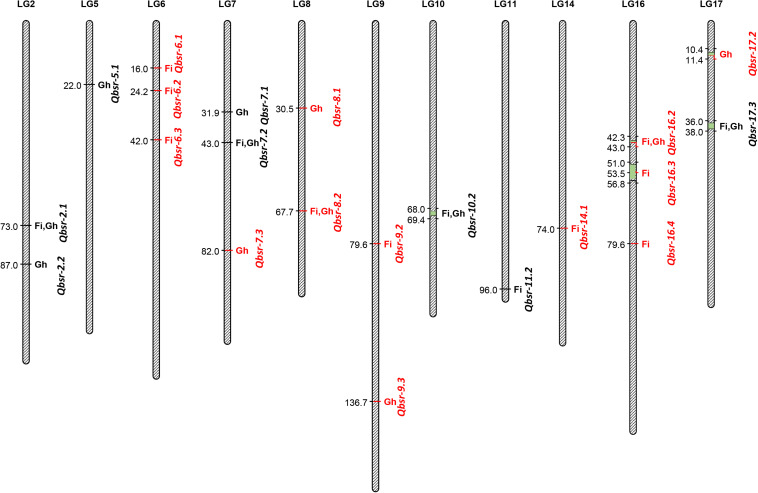
Quantitative trait loci (QTL) associated with *Sclerotinia* basal stalk rot (BSR) resistance identified in the HA 89/*H. argophyllus* advanced backcross population. The QTL with red fonts are from *H. argophyllus* and the QTL with black fonts are from HA 89. Fi, field; Gh, greenhouse.

A total of 21 QTL associated with BSR resistance were detected on 11 chromosomes, each explaining a proportion of the phenotypic variation ranging from 4.5 to 22.6% (Table 5 and Figure 2). Thirteen of these QTL had resistance alleles derived from the wild *H. argophyllus*, while the remaining eight QTL had positive alleles derived from the susceptible recurrent parent, HA 89. The highest number of QTL was detected on LGs 6, 7, and 16 with three QTL each, followed by LGs 2, 8, 9, and 17 with two QTL each. A total of eight QTL, *Qbsr-6.1*, *Qbsr-6.2*, *Qbsr-6.3*, *Qbsr-9.2*, *Qbsr-11.2*, *Qbsr-14.1*, *Qbsr-16.3*, and *Qbsr-16.4*, were detected for BSR DI measured in the field; seven QTL, *Qbsr-2.2*, *Qbsr-5.1*, *Qbsr-7.1*, *Qbsr-7.3*, *Qbsr-8.1*, *Qbsr-9.3*, and *Qbsr-17.2*, were detected for the traits measured in the greenhouse, while six QTL, *Qbsr-2.1*, *Qbsr-7.2*, *Qbsr-8.2*, *Qbsr-10.2*, *Qbsr-16.2*, and *Qbsr-17.3*, were detected from both field and greenhouse tests (Table 5). A detailed description of the QTL identification in individual screening tests and in combined analysis is presented in Supplementary Table 3. In the *Qbsr-17.3* genomic region, a total of four individual environmental QTL were detected within a 2-cM span of LG17 (Supplementary Table 3). Three individual environment/combined analysis QTL were detected in the genomic regions of each of the following QTL: *Qbsr-7.2*, *Qbsr-8.2*, *Qbsr-14.1*, *Qbsr-16.2*, and *Qbsr-16.4*. In the *Qbsr-6.2* and *Qbsr-11.1* genomic regions, BSR DI QTL were detected only in the 2017 and 2018 field screening tests, respectively, but with high LOD values in multiple software packages. The remaining 13 QTL genomic regions had QTL detected in two analyses (Supplementary Table 3).

**TABLE 5:** Summary of *Sclerotinia* basal stalk rot resistance QTL identified in the HA 89/*H. argophyllus* AB-QTL population.

QTL	Environment	Linkage group	Position (cM)	LOD range	Flanking markers		*R*^2^ range	Resistance allele source
					Left	Right		
*Qbsr-2.1*	Field, GH	2	73.0	4.45-6.01	C2_157528095	C2_174979149	10.5-14.3	HA 89
*Qbsr-2.2*	GH	2	87.0	5.86-4.39	C2_141062679	C2_107610652	10.2-10.9	HA 89
*Qbsr-5.1*	GH	5	22.0	4.53-5.33	C5_44061240	C5_55959009	14.4-15.0	HA 89
*Qbsr-6.1*	Field	6	16.0	7.14-12.49	C6_6832430	C6_5701209	10.8-19.5	*H. argophyllus*
*Qbsr-6.2*	Field	6	24.2	7.78	C6_78289542	C6_83474175	17.3	*H. argophyllus*
*Qbsr-6.3*	Field	6	42.0	6.47-10.75	C6_5702968	C6_6001429	7.8-12.4	*H. argophyllus*
*Qbsr-7.1*	GH	7	31.9	5.10-5.80	C7_56631029	C7_33687882	12.2-13.9	HA 89
*Qbsr-7.2*	Field, GH	7	43.0	4.81-9.55	C7_62465916	C7_62041373	4.5-7.5	HA 89
*Qbsr-7.3*	GH	7	82.0	4.93-15.78	C7_62500940	C7_86188878	6.1-7.1	*H. argophyllus*
*Qbsr-8.1*	GH	8	30.5	4.28-6.67	C8_55328277	C8_66678828	16.7-19.7	*H. argophyllus*
*Qbsr-8.2*	Field, GH	8	67.7	5.44-8.77	C8_65495308	C8_71401840	6.7-14.3	*H. argophyllus*
*Qbsr-9.2*	Field	9	79.6	5.02-5.51	C9_176724553	C9_176603426	13.5-18.3	*H. argophyllus*
*Qbsr-9.3*	GH	9	136.7	5.028-9.76	C9_110189180	C9_173918742	14.8-17.3	*H. argophyllus*
*Qbsr-10.2*	Field, GH	10	68.0-69.4	5.58-6.33	C10_180847742	C10_111029421	10.5-16.3	HA 89
*Qbsr-11.2*	Field	11	96.0	12.28	C11_26402705	C11_39839617	11.3	HA 89
*Qbsr-14.1*	Field	14	73.9-74.0	3.34-6.83	C14_38960647	C14_92847867	5.5-22.6	*H. argophyllus*
*Qbsr-16.2*	Field, GH	16	42.3-43.0	3.28-19.13	C16_156300872	C16_146472171	10.7-19.1	*H. argophyllus*
*Qbsr-16.3*	Field	16	51.0-56.8	4.04-6.37	C16_154054455	C16_146426614	3.14-16.9	*H. argophyllus*
*Qbsr-16.4*	Field	16	79.6	5.87-6.97	C16_75842994	C16_63009695	13.0-19.5	*H. argophyllus*
*Qbsr-17.2*	GH	17	10.4-11.4	3.11-6.10	C17_99950806	C17_68503520	17.0-17.3	*H. argophyllus*
*Qbsr-17.3*	Field, GH	17	36.0-38.0	3.35-13.94	C17_171247454	C17_174047539	8.7-19.4	HA 89

*LOD, log of odds; *R*^2^, phenotypic variation explained; GH, greenhouse.*

## Discussion

Sunflower was originally domesticated in the United States and its CWR have co-evolved with biotic and abiotic stresses in their habitat and, thus, potentially possess genes for desired traits (Seiler et al., 2017). Several sunflower CWR have been reported as potential sources of *S. sclerotiorum* resistance (Henn et al., 1997; Cerboncini et al., 2002; Rönicke et al., 2004; Block et al., 2008, 2009, 2010; Jan et al., 2008; Seiler, 2010). However, none of these studies pursued detailed analysis of *Sclerotinia* resistance with respect to their genetic position in the sunflower genome nor released any germplasm lines to be utilized in practical breeding programs until recently. Introgression of BSR resistance has been achieved from *H. argophyllus*, *H. petiolaris*, and *H. praecox* into the cultivated sunflower background (Qi et al., 2016b; Talukder et al., 2019a), and highly BSR-resistant introgression lines have been released for further use in sunflower breeding programs (Qi et al., 2018; Talukder et al., 2019b). In order to dissect the genetic basis of BSR resistance introgressed from *H. argophyllus*, we used an AB-QTL mapping population combined with GBS genotyping to identify 21 QTL associated with BSR resistance on 11 sunflower chromosomes in the present study, each explaining a phenotypic variation ranging from 4.5 to 22.6%. To our knowledge, this is the first report on mapping genomic regions responsible for the BSR resistance to *S. sclerotiorum* from sunflower wild species, which would increase the level of genetic diversity present in sunflowers to manage this pathogen. SNP markers flanking the QTL identified will facilitate marker-assisted selection of these critical resistances in sunflower breeding.

Field screening of the AB-QTL population showed low (2017) to moderate (2018 and 2019) levels of BSR DI, indicating considerable variation in plant host responses from even small changes in environmental conditions (Figure 1). Despite variable DI ranges in 3 years, the frequency distribution was continuous in all three seasons, confirming a quantitatively inherited trait. A similar distribution was also observed for the two traits, AUDPC and DR, which were measured in the greenhouse (Figure 1). The high disease pressure in the greenhouse screening was evident from the poor performance of the susceptible parent HA 89. Due to the growth habit and idiotype, we did not place the wild parent alongside its progeny lines in the field or in the greenhouse trials for the BSR response to observe any transgressive segregation. In the ANOVA analysis of the field data, significant variation for BSR DI was observed only for the genotype with no significant environment or G × E interaction for the trait in the population (Table 1). This was further supported by a moderately high (0.74) broad-sense heritability (*H*^2^) estimate, a measure which estimates the portion of observed phenotype that is due to genetic factors. The greenhouse screening trial also showed a similar level for the heritability estimates (0.73) for the traits.

The highly significant Spearman’s rank correlations (*ρ*) between BSR DI scores across years signify the linear relationship of genotypes in different environments as well as validate the repeatability of the field screening trials (Table 2). However, the relationship between the field and greenhouse screening trials was not significant except for AUDPC and DI of 2019, which was barely significant at *p* < 0.05. Poor correlation between greenhouse and field disease evaluations was somewhat unexpected as greenhouse BSR evaluations have previously been observed to correlate strongly with field observations in a panel of cultivated sunflower lines (Underwood et al., 2020). Resistance introgressed from *H. argophyllus* may involve novel mechanisms that are not captured in the greenhouse evaluation. Alternatively, some of the apparent resistance derived from *H. argophyllus* may be a consequence of disease avoidance in field trials due to differences in plant growth habit or time to flowering and maturity. Nonetheless, in spite of the poor correlation between field and greenhouse disease observations, six BSR resistance QTL were detected in both the field and greenhouse trials.

The high-density genetic map was made with 3,110 SNP markers developed using GBS technology in the AB-QTL population. The high level of DNA polymorphism between the two genetically diverse parental lines makes it possible to map such a large number of SNP markers. This is, so far, the densest linkage map developed using a single biparental mapping population in sunflower. The current linkage map spans the longest genetic distance (2,045.14 cM) over any other SNP marker-based linkage maps developed earlier in sunflower, where the previous longest map reported was 1,505.33 cM (Talukder et al., 2014a, 2020). This implies that the present molecular map thus has the largest relative coverage of the sunflower genome, which is important for mapping QTL that confer the economically important traits of this species.

We performed QTL analyses of the field DI data individually for each year as well as a combined analysis of 3 years of phenotype data using extracted best linear unbiased predictor (BLUP) values. The greenhouse test data were also analyzed independently. Altogether, we detected 21 QTL associated with BSR resistance in this population, which is by far the greatest number of BSR resistance QTL reported in a single sunflower mapping population. The majority of the QTL alleles (∼62%) conferring BSR resistance were derived from the wild *H. argophyllus* parent, which was expected as the wild parent showed much higher BSR resistance than the cultivated sunflower lines (Qi et al., 2016b). Out of the 21 QTL, only six were common in both field and greenhouse environments, while the remaining 15 QTL were detected independently, eight in the field and seven in the greenhouse. This result seems generally consistent with the poor correlation between the field and the greenhouse data (Table 2).

Significant BSR resistance QTL were detected in 11 of the 17 sunflower LGs in this *H. argophyllus* AB-QTL population (Figure 2). Except for LGs 5 and 7, BSR resistance QTL were previously reported in the remaining nine LGs—2, 6, 8, 9, 10, 14, 16, and 17 (Davar et al., 2010; Amouzadeh et al., 2013). However, due to the lack of common markers, as well as a lack of sequence information and/or physical position of the QTL flanking markers in earlier studies, a detailed comparison of the QTL position could not be performed with the current study. Talukder et al. (2016) reported two large-effect QTL, *Qbsr-10.1* and *Qbsr-17.1*, identified in multiple environments, one each on LGs 10 and 17 where we also detected QTL on these two LGs in the current study. However, a comprehensive sequence comparison of the flanking SNP markers suggests that the QTL positions of these two studies do not match to their positions on the sunflower physical map, suggesting novel QTL for the *H. argophyllus* AB-QTL population (data not shown). Nevertheless, the individual environment-specific QTL *Qbsr-9.1* on LG9 reported by Talukder et al. (2016) coincides with the physical position of the *Qbsr-9.3* QTL of the current study on the sunflower genome.

Although an earlier report showed a poor correlation between *Sclerotinia* HR and BSR (Talukder et al., 2014b), co-localization of BSR QTL was observed with significant SNP markers associated with HR disease resistance. For example, SNP117 on LG10, SNP125 on LG2, and SNP44 on LG6, significantly associated with *Sclerotinia* HR (Filippi et al., 2020), were co-localized with the *Qbsr-10.1*, *Qbrs-2.2*, and *Qbrs-6.1* of the current study, respectively.

The resistance against the necrotrophic fungus *S. sclerotiorum* in sunflower involves many genes and biochemical pathways whose roles in plant resistance system have yet to be understood (Mengiste, 2012). Introgression of *H. argophyllus* alleles associated with BSR resistance potentially offers new allelic diversity for the cultivated sunflower gene pool and will likely serve as a useful tool for breeding sunflower lines with higher resistance against this devastating pathogen.

## Data Availability Statement

The datasets presented in this study can be found in online repositories. The names of the repository/repositories and accession number(s) can be found below: [European Variation Archive (EVA)], (Accessions: Project: PRJEB41931 and Analyses: ERZ1691840).

## Author Contributions

LQ and ZT conceived and designed the experiments. ZT, WU, CM, GS, XL, XC, and LQ performed the experiments. ZT, LQ, and YL analyzed the data. ZT wrote the manuscript. LQ edited the manuscript. WU, GS, XL, XC, and YL commented on the manuscript before submission. All authors contributed to the article and approved the submitted version.

## Conflict of Interest

The authors declare that the research was conducted in the absence of any commercial or financial relationships that could be construed as a potential conflict of interest.

## Footnotes

1 https://www.heliagene.org/HanXRQ-SUNRISE

## Supplementary Material

Supplementary Figure 1 The panel shows the disease incidence of the parent, HA 89, the susceptible checks, Cargill 272, the resistant check, Croplan 305, and the 134 lines of the HA 89/*H. argophyllus* AB-QTL sunflower population evaluated for Sclerotinia basal stalk rot resistance in the field in Carrington, ND, United States during 2017 to 2019.

Supplementary Table 1 Flanking sequences (400 bp) of significant SNPs associated with BSR resistance detected in the HA 89/*H. argophyllus* advanced backcross population.

Supplementary Table 2 Detail of sunflower linkage map developed using SNP markers in the HA 89/*H. argophyllus* advanced backcross population. SNP markers with red fonts are co-segregating markers.

Supplementary Table 3 QTL associated with Sclerotinia basal stalk rot (BSR) disease incidence (DI), area under disease progress curve (AUDPC) and BSR disease rating (DR) in an advanced backcross population derived from the cross of HA 89 and wild annual sunflower species, *H. argophyllus* evaluated in the field and greenhouse.

## References

[B1] Amouzadeh M. Darvishzadeh R. Haddadi P. Abdollahi M. B. Rezaee D. Y. (2013). Genetic analysis of partial resistance to basal stem rot (*Sclerotinia sclerotiorum*) in sunflower. *Genetika* 45 737–748. 10.2298/GENSR1303737A

[B2] Badouin H. Gouzy J. Grassa C. J. Murat F. Staton S. E. Cottret L. (2017). The sunflower genome provides insights into oil metabolism, flowering and Asterid evolution. *Nature* 546 148–152. 10.1038/nature22380 28538728

[B3] Block C. C. Marek L. F. Gulya T. J. (2008). “Evaluation of wild *Helianthus* species for resistance to *Sclerotinia* stalk rot,” in *Proceedings of the 6th Annual Sclerotinia Initiative Meeting*, Bloomington, MN.

[B4] Block C. C. Marek L. F. Gulya T. J. (2009). “Evaluation of wild *Helianthus* species for resistance to *Sclerotinia* stalk rot,” in *Proceedings of the 7th Annual Sclerotinia Initiative Meeting*, Bloomington, MN.

[B5] Block C. C. Marek L. F. Gulya T. J. (2010). “Evaluation of wild *Helianthus* species for resistance to *Sclerotinia* stalk rot,” in *Proceedings of the 8th Annual Sclerotinia Initiative Meeting*, Bloomington, MN.

[B6] Boland G. Hall R. (1994). Index of plant hosts of *Sclerotinia sclerotiorum*. *Can. J. Plant Pathol.* 16 93–108. 10.1080/07060669409500766

[B7] Box G. E. Cox D. R. (1964). An analysis of transformations. *J. R. Stat. Soc. Ser. B Stat. Methodol.* 26 211–243.

[B8] Cerboncini C. Beine G. Binsfeld P. Dresen B. Peisker H. Zerwas A. (2002). Sources of resistance to *Sclerotinia sclerotiorum* (Lib.) de Bary in a natural *Helianthus* gene pool. *Helia* 25 167–176. 10.2298/HEL0236167C

[B9] Chen J. F. Hu J. G. Vick B. A. Jan C. C. (2006). Molecular mapping of a nuclear male-sterility gene in sunflower (*Helianthus annuus* L.) using TRAP and SSR markers. *Theor. Appl. Genet.* 113 122–127. 10.1007/s00122-006-0278-2 16614829

[B10] Churchill G. A. Doerge R. W. (1994). Empirical threshold values for quantitative trait mapping. *Genetics* 138 963–971. 10.1093/genetics/138.3.963 7851788 PMC1206241

[B11] Davar R. Darvishzadeh R. Majd A. Ghosta Y. Sarrafi A. (2010). QTL mapping of partial resistance to basal stem rot in sunflower using recombinant inbred lines. *Phytopathol. Mediterr.* 49 330–341. 10.14601/Phytopathol_Mediterr-8374

[B12] Elshire R. J. Glaubitz J. C. Sun Q. Poland J. A. Kawamoto K. Buckler E. S. (2011). A robust, simple genotyping-by-sequencing (GBS) approach for high diversity species. *PLoS One* 6:e19379. 10.1371/journal.pone.0019379 21573248 PMC3087801

[B13] FAOSTAT (2020). *Food and Agriculture Organization of United Nations.* Rome: FAO.

[B14] Filippi C. V. Zubrzycki J. E. Di Rienzo J. A. Quiroz F. J. Puebla A. F. Alvarez D. C. (2020). Unveiling the genetic basis of *Sclerotinia* head rot resistance in sunflower. *BMC Plant Biol.* 20:322. 10.1186/s12870-020-02529-7 32641108 PMC7346337

[B15] Glaubitz J. C. Casstevens T. M. Lu F. Harriman J. Elshire R. J. Sun Q. (2014). TASSEL-GBS: a high capacity genotyping by sequencing analysis pipeline. *PLoS One* 9:e90346. 10.1371/journal.pone.0090346 24587335 PMC3938676

[B16] Gulya T. Harveson R. Mathew F. Block C. Thompson S. Kandel H. (2019). Comprehensive disease survey of US sunflower: disease trends, research priorities and unanticipated impacts. *Plant Dis.* 103 601–618. 10.1094/pdis-06-18-0980-fe 30789318

[B17] Gulya T. J. Radi S. Balbyshev N. (2008). “Large scale field evaluations for *Sclerotinia* stalk rot resistance in cultivated sunflower,” in *Proceedings of the 17th International Sunflower Conference*, ed. Velasco L. (Paris: International Sunflower Association), 175–179.

[B18] Gulya T. J. Rashid K. Y. Masirevic S. M. (1997). “Sunflower diseases,” in *Sunflower Technology and Production*, ed. Schneiter A. A. (Madison, WI: ASA-CSSA-SSSA), 263–379. 10.2134/agronmonogr35.c6

[B19] Henn H.-J. Steiner U. Wingender R. Schnabl H. (1997). Wildtype sunflower clones: source for resistance against *Sclerotinia sclerotiorum* (Lib.) de Bary stem infection. *Angew. Bot.* 71 5–9.

[B20] Horne E. C. Kumpatla S. P. Patterson K. A. Gupta M. Thompson S. A. (2004). Improved high-throughput sunflower and cotton genomic DNA extraction and PCR fidelity. *Plant Mol. Biol. Rep.* 22 83–84. 10.1007/BF02773352

[B21] Hubner S. Bercovich N. Todesco M. Mandel J. R. Odenheimer J. Ziegler E. (2019). Sunflower pan-genome analysis shows that hybridization altered gene content and disease resistance. *Nat. Plants* 5 54–62. 10.1038/s41477-018-0329-0 30598532

[B22] Hulke B. S. Ma G. Qi L. L. Gulya T. J. (2018). Registration of oilseed sunflower germplasms RHA 461, RHA 462, RHA 463, HA 465, HA 466, HA 467, and RHA 468 with diversity in *Sclerotinia* resistance, yield, and other traits. *J. Plant Regist.* 12 142–147. 10.3198/jpr2017.04.0023crg

[B23] Jan C.-C. Rutger J. N. (1988). Mitomycin C- and Streptomycin-induced male sterility in cultivated sunflower. *Crop Sci.* 28 792–795. 10.2135/cropsci1988.0011183X002800050014x

[B24] Jan C.-C. Seiler G. J. Gulya T. J. Feng J. (2008). “Sunflower germplasm development utilizing wild *Helianthus* species,” in *Proceedings of the 17th International Sunflower Conference*, ed. Velasco L. (Paris: International Sunflower Association), 29–43.

[B25] Joehanes R. Nelson J. C. (2008). QGene 4.0, an extensible Java QTL-analysis platform. *Bioinformatics* 24 2788–2789. 10.1093/bioinformatics/btn523 18940826

[B26] Kosambi D. D. (1943). The estimation of map distances from recombination values. *Ann. Eugen.* 12 172–175. 10.1111/j.1469-1809.1943.tb02321.x

[B27] Langmead B. Salzberg S. L. (2012). Fast gapped-read alignment with Bowtie 2. *Nat. Methods* 9 357–359. 10.1038/nmeth.1923 22388286 PMC3322381

[B28] Ma G. J. Markell S. G. Song Q. J. Qi L. L. (2017). Genotyping-by-sequencing targeting of a novel downy mildew resistance gene *Pl*_20_ from wild *Helianthus argophyllus* for sunflower (*Helianthus annuus* L.). *Theor. Appl. Genet.* 130 1519–1529. 10.1007/s00122-017-2906-4 28432412

[B29] Masirevic S. Gulya T. J. (1992). *Sclerotinia* and *Phomopsis* - two devastating sunflower pathogens. *Field Crops Res.* 30 271–300. 10.1016/0378-4290(92)90004-S

[B30] Meng L. Li H. Zhang L. Wang J. (2015). QTL IciMapping: integrated software for genetic linkage map construction and quantitative trait locus mapping in biparental populations. *Crop J.* 3 269–283. 10.1016/j.cj.2015.01.001

[B31] Mengiste T. (2012). Plant immunity to necrotrophs. *Annu. Rev. Phytopathol.* 50 267–294. 10.1146/annurev-phyto-081211-172955 22726121

[B32] Miller J. F. Gulya T. J. (1999). Registration of eight *Sclerotinia*-tolerant sunflower germplasm lines. *Crop Sci.* 39 301–302. 10.2135/cropsci1999.0011183X003900010075x

[B33] Miller J. F. Gulya T. J. Vick B. A. (2006). Registration of two maintainer (HA 451 and HA 452) and three restorer (RHA 453-RHA 455) *Sclerotinia*-tolerant oilseed sunflower germplasms. *Crop Sci.* 46 2727–2728. 10.2135/cropsci2006.06.0436

[B34] Money K. L. Koehler B. D. Misar C. G. Grove M. Underwood W. Hulke B. S. (2019). Registration of oilseed sunflower germplasms RHA 485, RHA 486, and HA 487, selected for resistance to *Phomopsis* stalk canker and *Sclerotinia*, in a high-yielding and high-oil background. *J. Plant Regist.* 13 439–442. 10.3198/jpr2019.02.0008crg

[B35] Nyquist W. E. (1991). Estimation of heritability and prediction of selection response in plant populations. *Crit. Rev. Plant Sci.* 10 235–322. 10.1080/07352689109382313

[B36] Qi L. L. Foley M. E. Cai X. W. Gulya T. J. (2016a). Genetics and mapping of a novel downy mildew resistance gene, *Pl*_18_, introgressed from wild *Helianthus argophyllus* into cultivated sunflower (*Helianthus annuus* L.). *Theor. Appl. Genet.* 129 741–752. 10.1007/s00122-015-2662-2 26747047

[B37] Qi L. L. Long Y. Talukder Z. I. Seiler G. J. Block C. C. Gulya T. J. (2016b). Genotyping-by-sequencing uncovers the introgression alien segments associated with *Sclerotinia* basal stalk rot resistance from wild species-I. *Helianthus argophyllus* and *H. petiolaris*. *Front. Genet.* 7:219. 10.3389/fgene.2016.00219 28083014 PMC5183654

[B38] Qi L. L. Talukder Z. I. Long Y. M. Seiler G. J. (2018). Registration of oilseed sunflower germplasms HA-BSR2, HA-BSR3, HA-BSR4, and HA-BSR5 with resistance to *Sclerotinia* basal stalk rot and downy mildew. *J. Plant Regist.* 12 399–404. 10.3198/jpr2017.11.0083crg

[B39] R Core Team (2017). *R: A Language and Environment for Statistical Computing, v 3.4.3.* Vienna: R Foundation for Statistical Computing.

[B40] Rönicke S. Hahn V. Horn R. Grone I. Brahm L. Schnabl H. (2004). Interspecific hybrids of sunflower as a source of *Sclerotinia* resistance. *Plant Breed.* 123 152–157. 10.1046/j.1439-0523.2003.00925.x

[B41] SAS Institute (2016). *The SAS System for Windows, v 9.4.* Cary, NC: SAS Institute Inc.

[B42] Schneiter A. A. Miller J. F. (1981). Description of sunflower growth stages. *Crop Sci.* 21 901–903. 10.2135/cropsci1981.0011183X002100060024x

[B43] Seiler G. J. (2010). “Utilization of wild *Helianthus* species in breeding for disease resistance,” in *Proceedings of the International Symposium “Sunflower Breeding on Resistance to Diseases*, Paris.

[B44] Seiler G. J. Qi L. L. Marek L. F. (2017). Utilization of sunflower crop wild relatives for cultivated sunflower improvement. *Crop Sci.* 57 1083–1101. 10.2135/cropsci2016.10.0856

[B45] Shaner G. Finney R. E. (1977). The effect of nitrogen fertilization on the expression of slow-mildewing resistance in Knox wheat. *Phytopathology* 67 1051–1056. 10.1094/Phyto-67-1051 40934422

[B46] Smart B. C. Koehler B. D. Misar C. G. Gulya T. J. Hulke B. S. (2019). Registration of oilseed sunflower germplasms HA 482, RHA 483, and RHA 484 selected for resistance to *Sclerotinia* and *Phomopsis* diseases. *J. Plant Regist.* 13 450–454. 10.3198/jpr2019.07.0030crg

[B47] Stam P. (1993). Construction of integrated genetic linkage maps by means of a new computer package: join Map. *Plant J.* 3 739–744. 10.1111/j.1365-313X.1993.00739.x

[B48] Talukder Z. I. Gong L. Hulke B. S. Pegadaraju V. Song Q. Schultz Q. (2014a). A high-density SNP map of sunflower derived from RAD-sequencing facilitating fine-mapping of the rust resistance gene *R*_12_. *PLoS One* 9:e98628. 10.1371/journal.pone.0098628 25014030 PMC4094432

[B49] Talukder Z. I. Hulke B. S. Marek L. F. Gulya T. J. (2014b). Sources of resistance to sunflower diseases in a global collection of domesticated USDA plant introductions. *Crop Sci.* 54 694–705. 10.2135/cropsci2013.07.0506

[B50] Talukder Z. I. Hulke B. S. Qi L. L. Scheffler B. E. Pegadaraju V. McPhee K. (2014c). Candidate gene association mapping of *Sclerotinia* stalk rot resistance in sunflower (*Helianthus annuus* L.) uncovers the importance of *COI*1 homologs. *Theor. Appl. Genet.* 127 193–209. 10.1007/s00122-013-2210-x 24193356

[B51] Talukder Z. I. Hu J. Seiler G. J. Qi L. L. (2017). Registration of oilseed sunflower germplasm HA-BSR1 highly tolerant to *Sclerotinia* basal stalk rot. *J. Plant Regist.* 11 315–319. 10.3198/jpr2016.10.0060crg

[B52] Talukder Z. I. Long Y. M. Seiler G. J. Underwood W. Qi L. L. (2019a). Introgression and monitoring of wild *Helianthus praecox* alien segments associated with *Sclerotinia* basal stalk rot resistance in sunflower using genotyping-by-sequencing. *PLoS One* 14:e0213065. 10.1371/journal.pone.0213065 30822322 PMC6396933

[B53] Talukder Z. I. Long Y. M. Seiler G. J. Underwood W. Qi L. L. (2019b). Registration of oilseed sunflower germplasms HA-BSR6, HA-BSR7, and HA-BSR8 highly resistant to *Sclerotinia* basal stalk rot and downy mildew. *J. Plant Regist.* 13 433–438. 10.3198/jpr2018.10.0071crg

[B54] Talukder Z. I. Seiler G. J. Song Q. Ma G. Qi L. L. (2016). SNP discovery and QTL mapping of *Sclerotinia* basal stalk rot resistance in sunflower using genotyping-by-sequencing. *Plant Genome* 9:35. 10.3835/plantgenome2016.03.0035 27902793

[B55] Talukder Z. I. Underwood W. Ma G. Seiler G. J. Misar C. G. Cai X. (2020). Genetic dissection of *Phomopsis* stem canker resistance in cultivated sunflower using high density SNP linkage map. *Int. J. Mol. Sci.* 21:1497. 10.3390/ijms21041497 32098308 PMC7073018

[B56] Tanksley S. Nelson J. (1996). Advanced backcross QTL analysis: a method for the simultaneous discovery and transfer of valuable QTLs from unadapted germplasm into elite breeding lines. *Theor. Appl. Genet.* 92 191–203. 10.1007/bf00223376 24166168

[B57] Underwood W. Misar C. G. Block C. C. Gulya T. J. Talukder Z. I. Hulke B. S. (2020). A greenhouse method to evaluate sunflower quantitative resistance to basal stalk rot caused by *Sclerotinia sclerotiorum*. *Plant Dis.* 10.1094/pdis-08-19-1790-re 33264029

[B58] USDA (2020). *Oilseeds: World Markets and Trade.* Washington, DC: USDA.

[B59] Utz H. F. Melchinger A. E. (2006). *PLABQTL: A Computer Program to Map QTL, v 1.2. Institute of Plant Breeding, Seed Science, and Population Genetics.* Stuttgart: Univeristy of Hohenheim.

[B60] Van Ooijen J. (2006). *JoinMap^®^ 4, Software for the Calculation of Genetic Linkage Maps in Experimental Populations.* Wageningen: Kyazma BV.

[B61] Venables W. N. Ripley B. D. (2002). *Modern Applied Statistics with S.* New York, NY: Springer-Verlag.

[B62] Voorrips R. E. (2002). MapChart: software for the graphical presentation of linkage maps and QTLs. *J. Hered.* 93 77–78. 10.1093/jhered/93.1.77 12011185

[B63] Wang S. Basten C. J. Zeng Z. B. (2012). *Windows QTL Cartographer, v 2.5.* Raleigh, NC: Department of Statistics, North Carolina State University.

[B64] Zeng Z. B. (1994). Precision mapping of quantitative trait loci. *Genetics* 136 1457–1468. 10.1093/genetics/136.4.1457 8013918 PMC1205924

