# Supplementary figures and images for: Unraveling the Sclerotinia Basal Stalk Rot Resistance Derived From Wild Helianthus argophyllus Using a High-Density Single Nucleotide Polymorphism Linkage Map
Source: Front Plant Sci. 2021 Feb 3;11:617920. doi: 10.3389/fpls.2020.617920 (PMC7886805; doi:10.3389/fpls.2020.617920)

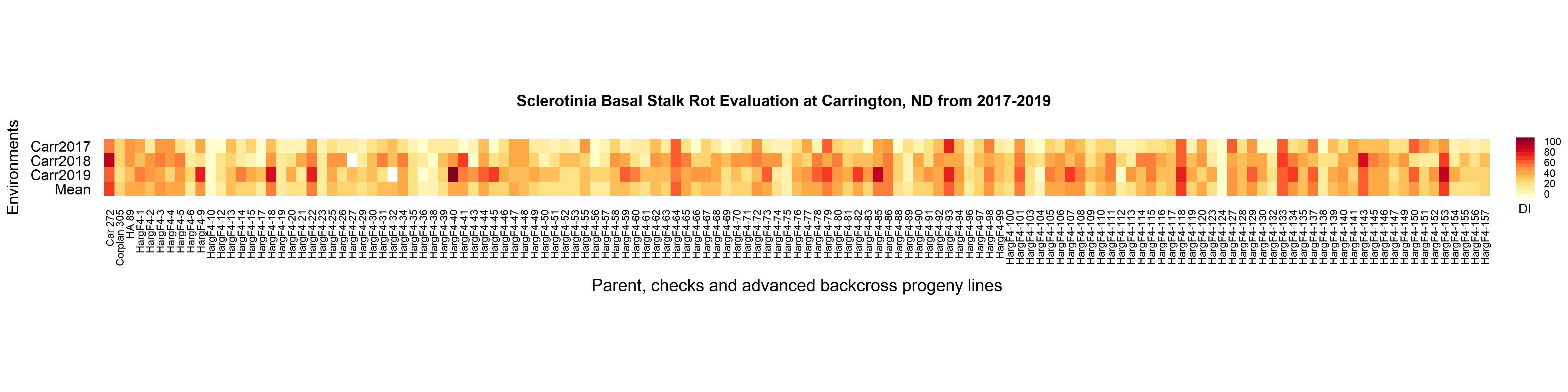

Supplement: Supplementary Figure 1 — The panel shows the disease incidence of the parent, HA 89, the susceptible checks, Cargill 272, the resistant check, Croplan 305, and the 134 lines of the HA 89/H. argophyllus AB-QTL sunflower population evaluated for Sclerotinia basal stalk rot resistance in the field in Carrington, ND, United States during 2017 to 2019. [file Image_1.TIFF]
